# The Daalbirrwirr Gamambigu (Safe Children) Model: Embedding Cultural Safety in Child Protection Responses for Australian Aboriginal Children in Hospital Settings

**DOI:** 10.3390/ijerph19095381

**Published:** 2022-04-28

**Authors:** Tara Flemington, Jennifer Fraser, Clinton Gibbs, Joanne Shipp, Joe Bryant, Amanda Ryan, Devika Wijetilaka, Susan Marks, Mick Scarcella, Dimitra Tzioumi, Shanthi Ramanathan, Liesa Clague, Donna Hartz, Bob Lonne, Mark Lock (Ngiyampaa)

**Affiliations:** 1 Nursing, Midwifery and Service Reform, Mid North Coast Local Health District, Coffs Harbour, NSW 2450, Australia; tara.flemington@health.nsw.gov.au; 2 Faculty of Medicine and Health, Susan Wakil School of Nusing and Midwifery, University of Sydney, Camperdown, NSW 2006, Australia; 3 Nursing, Midwifery and Education, The Sydney Children’s Hospitals Network, Westmead, NSW 2145, Australia; 4 Health Reform, Opportunities and Transition, Mid North Coast Local Health District, Port Macquarie, NSW 2444, Australia; clinton.gibbs@health.nsw.gov.au; 5 Integrated Child, Youth and Family Wellbeing, Mid North Coast Local Health District, Port Macquarie, NSW 2444, Australia; joanne.shipp@health.nsw.gov.au; 6 Aboriginal Health Strategy Unit, Mid North Coast Local Health District, Coffs Harbour, NSW 2450, Australia; joseph.bryant@health.nsw.gov.au; 7 Aboriginal Health Strategy Unit, Mid North Coast Local Health District, Port Macquarie, NSW 2444, Australia; amanda.ryan@health.nsw.gov.au; 8 Paediatrics, Mid North Coast Local Health District, Coffs Harbour, NSW 2450, Australia; devika.wijetilaka@health.nsw.gov.au; 9 Child Protection Unit, The Sydney Children’s Hospitals Network, Westmead, NSW 2145, Australia; susan.marks@health.nsw.gov.au; 10 Aboriginal Health, The Sydney Children’s Hospitals Network, Westmead, NSW 2145, Australia; michele.scarcella@health.nsw.gov.au; 11 Child Protection Unit, The Sydney Children’s Hospitals Network, Randwick, NSW 2031, Australia; dimitra.tzioumi@health.nsw.gov.au; 12 Child Protection and Wellbeing, Ministry of Health, St Leonards, NSW 2065, Australia; 13 Faculty of Medicine, School of Women’s and Children’s Health, University of New South Wales, Kensington, NSW 2052, Australia; 14 Health Research Economics, Hunter Medical Research Institute, New Lambton Heights, NSW 2305, Australia; shanthi.ramanathan@hmri.org.au; 15 College of Health, Medicine and Wellbeing, University of Newcastle, Callaghan, NSW 2308, Australia; 16 School of Nursing, Midwifery, Health Science and Physiotherapy, The University of Notre Dame, Darlinghurst, NSW 2010, Australia; liesa.clague@nd.edu.au; 17 School of Nursing and Midwifery, College of Medicine Health & Wellbeing, University of Newcastle, Gosford, NSW 2250, Australia; donna.hartz@newcastle.edu.au; 18 School of Public Health and Social Work, Faculty of Health, Queensland University of Technology, Kelvin Grove, QLD 4059, Australia; b.lonne@qut.edu.au; 19 Faculty of Health, School of Public Health, University of Technology Sydney, Ultimo, NSW 2007, Australia; mark.lock@uts.edu.au

**Keywords:** child protection, healthcare, aboriginal, cultural safety, critical consciousness, interprofessional collaboration, translational research, Australia, model of care, hospital, paediatric, emergency department

## Abstract

The aim of this paper is to describe the development of a model of care to embed cultural safety for Aboriginal children into paediatric hospital settings. The Daalbirrwirr Gamambigu (pronounced “Dahl-beer-weer gum-um-be-goo” in the Gumbaynggirr language means ‘safe children’) model encompasses child protection responses at clinical, managerial and organisational levels of health services. A review of scholarly articles and grey literature followed by qualitative interviews with Aboriginal health professionals formed the evidence base for the model, which then underwent rounds of consultation for cultural suitability and clinical utility. Culturally appropriate communication with children and their families using clinical yarning and a culturally adapted version of ISBAR (a mnemonic for Identify, Situation, Background, Assessment and Recommendation) for interprofessional communication is recommended. The model guides the development of a critical consciousness about cultural safety in health care settings, and privileges the cultural voices of many diverse Aboriginal peoples. When adapted appropriately for local clinical and cultural contexts, it will contribute to a patient journey experience of respect, dignity and empowerment.

## 1. Introduction

The cultural strengths of Aboriginal and Torres Strait Islander (hereafter and with respect, Aboriginal Australians) families resonate from 60,000 year-old cultures [1]. Aboriginal cultural practices help children to: (a) contribute to family, community and society; (b) develop independence, problem solving ability and decision-making; (c) understand relationships within the family and with Elders; (d) imbue a strong sense of cultural identity; and (e) contribute to stable and supportive environments [2,3]. This in turn promotes good physical health, as well as social, cultural, and emotional wellbeing [4].

For Australian health professionals, a culturally safe practice may be defined as ‘the ongoing critical reflection of health practitioner knowledge, skills, attitudes, practising behaviours and power differentials in delivering safe, accessible and responsive healthcare free of racism’ [5]. Against this background, stakeholders in hospital settings challenged us to answer the question: What does cultural safety look like and how do we do it in practice?

In 1997 the Bringing Them Home Report chronicled the policies and practices of Australian governments that led to the ‘Stolen Generation’; the widespread removal of Aboriginal children from their families, and its profound impacts on many children, families and communities [6]. The impacts include the physical, sexual and emotional maltreatment of children who were placed into institutional and foster care, and the resultant inter-generational trauma that resulted for many people, including stolen cultural identity [7]. The policy framework driving these removals reflected the systemic racism that was longstanding within the society and its political and social institutions, and was so pervasive that the Report found it to constitute “genocide”. Since 2000, the numbers of children in out-of-home-care have been increasing rapidly, with the rates for Aboriginal children leading the way. A recent report on Aboriginal children in the out-of-home care system identified a range of historical and contemporary issues including non-compliance with the Aboriginal Child Placement Principle (prioritising placements of children with kin), an increasing propensity to remove children at birth, inadequate use of preventive strategies and earlier intervention aimed at working with families, and placement instability and lack of suitable care providers due to existing procedures [8]. In 2008, the Australian Prime Minister and all state and federal governments gave public apologies which led to the Closing the Gap initiatives to redress the now-recognised gross inequalities in health, economic and social outcomes for Aboriginal Australians.

The extraordinary resilience of Aboriginal Australians—people belonging to communities with a deeply emotional history of cultural destruction—continues to be tested, resulting in intergenerational trauma and widespread disadvantage [7]. Since the first days of European occupation of Australia in the late 18th century, Aboriginal children have been forcibly removed from their families and communities, and hospitals have played a key part in this [9]. Initially, these practices were not based on an assessment of risk to the child and there was no requirement to provide evidence of maltreatment to justify the separation of children from families [10]. Indeed, child welfare laws (which required evidence of maltreatment to justify removal) did not apply to Aboriginal children until the 1940s. Since the introduction of these laws it has been necessary for child removal authorities to prove neglect and/or abuse—a somewhat arbitrary presentation in disadvantaged communities where poverty can present as neglect, particularly in the eyes of someone from outside the community, and someone who is not in a position to fully appreciate the social and emotional support network surrounding a child. Stereotyping and disapproval of Aboriginal Australians’ parenting practices have led to disastrous child protection responses—even when applied in good faith [10].

While the associations between social inequalities and health outcomes have long been recognised, research on the links that social inequalities and health outcomes have on child protection contact and interventions has not been the subject of detailed examination until relatively recently [11,12,13]. Perhaps unsurprisingly, in child protection systems around the globe, there is over-representation of children and families who are of colour, who are First Nations and who live in poverty [14,15,16,17]. Intersectionality occurs across a variety of factors, but what is clear is that historical policies including the removal of First Nations children and their placement away from their kin, culture and communities has resulted in longstanding and widespread fear of engagement with child protection authorities [17,18]. Despite system reforms and policy changes, these disproportionalities remain, and as a result movements are increasing around the globe toward public health approaches that prioritise earlier intervention and prevention strategies through universal services, including hospitals and health services [19,20,21]. This may be in response to the increasing recognition that public health approaches aim to address the underlying inequities that contribute to child maltreatment [20].

A public health approach to child protection has been promoted in Australia [19] but remains aspirational. This approach holds promise for addressing the rates of removal for Aboriginal children, which remain extraordinarily high. As of June 2020, there were 18,900 Aboriginal children in out-of-home-care, at a rate of 56 per 1000 children [22]. This is 11 times the rate for non-Aboriginal children, and while admissions of non-Aboriginal children to out-of-home-care have stabilized, the rate for Aboriginal children is increasing [22]. Over-representation of Australian Aboriginal children in child protection notifications and out-of-home care services have prevailed despite policies aimed at addressing this issue [23]. Furthermore, reports for neglect dominate [24], reflecting the long history of economic and social disadvantage experienced by Aboriginal Australians.

This is coupled with poor health outcomes for children in out-of-home care in general [25,26] and also for Aboriginal children [27,28]. For example, a Victorian health service reported widespread mental health problems (66%), hearing problems (37%), vision problems (34%), dental caries (40%) and developmental delays (46%) in a cohort of 103 Aboriginal children in out-of-home-care [29]. Medical and health personnel are the third highest source of notifications for potential abuse or neglect (12.5%) compared to school personnel (18.9%) and police (21.7%) [30]. Disproportionalities in child protection substantiations and admissions to care exacerbate existing health inequalities and, therefore, a public health approach to child protection calls for “interagency collaboration to address poor health and social outcomes of children” [31].

Descriptions of the significance of culture in this field are evident in research findings in general hospital care [32,33], child protection services delivery [34], child protection interprofessional relationships [35], the broader service delivery environment [36], in hospitals generally [37], and in emergency departments [38]. In health care and child protection, clinical safety is intextricably linked with cultural safety [5,39], although this has not been empirically investigated. In fact, few studies have examined how to successfully implement the necessary elements of cultural safety at the point of care [33].

Our primary goal was to produce a practical guide for clinicians that would be supported by an overarching and integrated organisational framework with measurable indicators of success. This paper describes the formative research processes and procedures for methodological and cultural rigor to develop a model that would address these inequalities in child protection and health outcomes for Aboriginal children.

## 2. Materials and Methods

We first present the settings and context of this project. This is followed by detailing the five project phases and the measures undertaken to ensure cultural leadership and cultural safety. These phases of the Model of Care (the model) development are:

- Developing appropriate governance and oversight;
- Cultural engagement process and mapping;
- Scoping literature review;
- Yarning groups and in-depth interviews; and
- Clinical utility testing.

### 2.1. Settings and Context

This formative research project was undertaken in the rural Mid North Coast and metropolitan Sydney areas of New South Wales, Australia, extending earlier translational child protection research in their hospitals. The translational research project, Maam-darrundaygam Daalbirrwirrr Gamambigu (Embedding Cultural Safety in Health Professional Child Protection Responses for Aboriginal Children), arose from Aboriginal health staff alerting the non-Aboriginal research team to the urgent need for clinical and organisational resources to improve health professional child protection responses for at-risk Aboriginal children.

There are approximately 265,685 Aboriginal people residing in New South Wales, representing 3.4% of the total population [40]. Aboriginal children represent 11.8% of all children in the Mid North Coast Local Health District, and between 0.6 and 5.1% of children across Sydney [41]. While emergency department presentations for Aboriginal children in the Sydney Children’s Hospitals Network are proportionate with the population figures (2.2%), they are substantially higher in rural areas [41].

The project took place on the Birpai, Dunghutti, Gumbaynggirr and Nganyaywana Nations/language groups (Mid North Coast Local Health District), the Gadigal and Bidjigal peoples who traditionally occupied the Sydney coast at Randwick, the Burramattagal peoples at Westmead, the Gayamaygal people of Manly and the Dharug people at Bankstown. We acknowledge that the Aboriginal participants in this project, while residing on these lands, are from many First Nations in Australia. We also acknowledge Aboriginal people as the traditional custodians of the lands on which this project took place, and their ongoing spiritual and cultural connections to country.

### 2.2. Phase 1: Developing Appropriate Governance and Oversight

Aboriginal Australians’ voices infused the methodology, which was framed by key documents that are significant in the institutional discourse of research, policy and practice with Aboriginal Australians. Firstly, the ethical principles of spirit and integrity, cultural continuity, equity, reciprocity, respect, and responsibility [42] guided the study design and ethics application through the NSW Aboriginal Health and Medical Research Council [43] (Aboriginal Health and Medical Research Council HREC: Ref 1369/18; Sydney Children’s Hospitals Network HREC: Ref LNR/17/SCHN/318). This protocol and the subsequent processes were then layered with the domains of the Cultural Respect Framework for Aboriginal and Torres Strait Islander Health [44]; the Ngaa-bi-nya Aboriginal and Torres Strait Program Evaluation Framework (hereafter, Ngaa-bi-nya Framework) [45]; the integrated Promoting Action on Research Implementation in Health Services (i-PARIHS) Framework [46]; and the building blocks of the Family Matters Roadmap (a national strategy to reduce the number of Aboriginal children removed from families) [39].

A culturally safe [47,48] research process was developed through the perspective of the cultural interface [49] by weaving [50] together a Wiradjuri worldview embedded in the Ngaa-bi-nya framework [45] and the Western world view embedded in the i-PARIHS framework [46]. The Ngaa-bi-nya framework (pronounced “naa-bi-nya”, which means to examine, try, and evaluate in the language of the Wiradjuri peoples) is one of few tools developed with Aboriginal worldviews of health [51], and it accounts for many of the factors that are relevant to Aboriginal people. It focusses on four domains—landscape factors, resources, ways of working and learnings. They in turn complement the i-PARIHS Framework domains of facilitation, innovation, recipients and context, as detailed elsewhere [33].

The project was led by a research team of seven Aboriginal and six non-Aboriginal investigators and supported by Aboriginal investigators with expertise in the field, aligned with cultural governance in research [52]. Operationally, the project team comprised of a non-Aboriginal post-doctoral project lead with qualifications in paediatric nursing, public health and child protection, and Aboriginal project officers with qualifications in health promotion/public health and allied health/Indigenous studies. Organisational governance was maintained by a project steering committee, of whom six members were Aboriginal, and represented disciplines of clinical governance, paediatric medicine, emergency medicine, child protection, social work, violence prevention, nursing, and midwifery, with executive representation from nursing, clinical governance and Aboriginal health (Appendix A).

### 2.3. Phase 2: Cultural Engagement Process and Mapping

The project team’s approach was further shaped by principles of research practice based on recognising the cultural world views of Aboriginal Australians [42,43,51], improving the cultural competency of non-Aboriginal peoples [53,54], promoting cultural safety in research processes [55], and “fostering cultural and emotional safety” [56] of Aboriginal research participants. This team ethic is evident in our consideration of reflexivity (Appendix A) and the team’s cultural diversity with Aboriginal (ML, JS, CG, JB, DH, MS and LC) and non-Aboriginal Australians (JF, TF, SR, BL, DT, SM) working together in the spirit of shared learning [47]. Additionally, Aboriginal people led the project’s governance, participated in clinical workshops, led workshops, conducted interviews, were involved in all aspects of data analysis, and wrote the reports and papers.

The team members developed a stakeholder cultural engagement map to ensure all relevant people were contacted, then invited for a yarn about the project, and formally invited to participate in accord with their own priorities. Feedback was welcomed on all aspects of the model, including visual presentation and design, evaluation measures, and clinical and cultural content. Stakeholders could provide feedback through a variety of channels (email, telephone or in-person) and to their choice of an Aboriginal or non-Aboriginal researcher.

**Figure 1:**
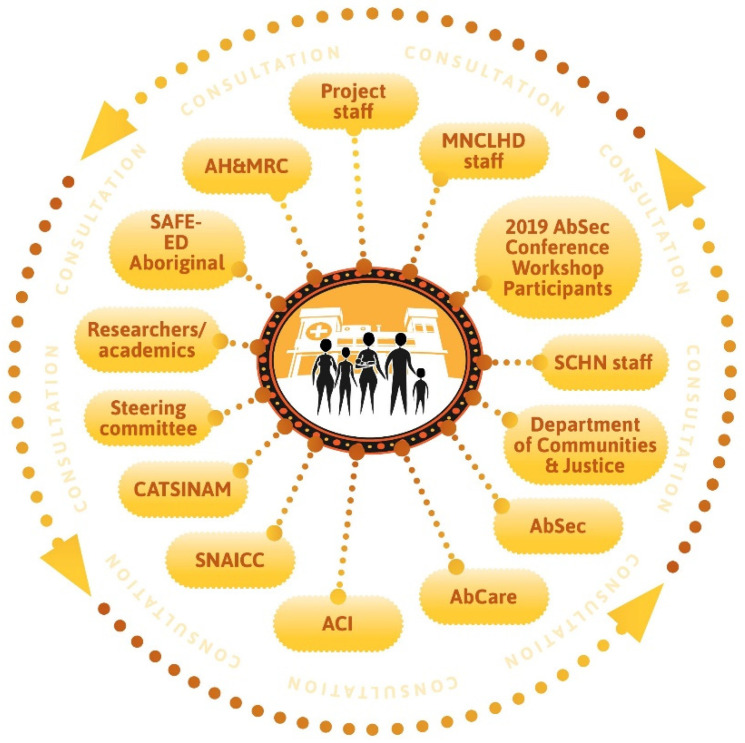
Daalbirrwirr Gamambigu Aboriginal consultation diagram.

The Daalbirrwirr Gamambigu Aboriginal Consultation Diagram (Figure 1) illustrates the range of stakeholders involved at the intersection of child protection and healthcare services. It presents consultation as a constant process instead of one-off engagement, creating wrap-around discourse between all the stakeholders (dotted circular line with arrows). Aboriginal families are located as the central focus of the model and service provision.

A key component of Aboriginal stakeholder testing took place at a public conference hosted by AbSec (the New South Wales Child, Family and Community Peak Aboriginal Corporation) [57]. The research team developed a workshop format that began with an opening presentation followed by three breakout sessions. Each group of 20 participants was guided by an Aboriginal team member to workshop one of three model components: the model thematic diagram; culturally appropriate approaches to interprofessional communication; and clinical yarning with families. The results of the one-hour long workshop were collated and analysed by the project team, then distributed to workshop participants afterwards for feedback and to demonstrate transparency (the workshop report is available on request).

Broader rounds of consultation and testing with Aboriginal stakeholders occurred throughout the model’s development with members of community organisations, nationally recognized peak Aboriginal professional organisations, professional networks and personal connections (Figure 1).

**Figure 2:**
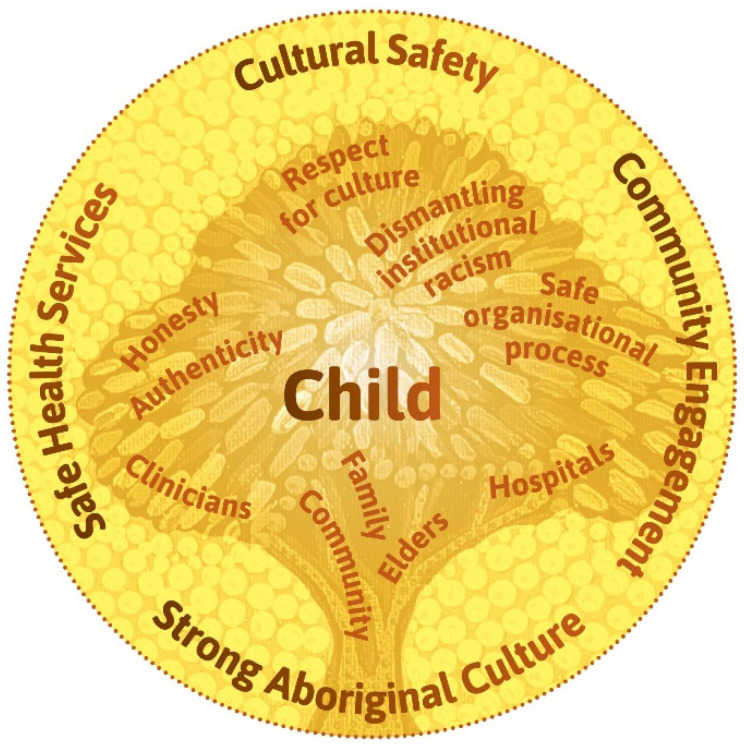
Daalbirrwirr Gamambigu thematic diagram.

The thematic diagram (Figure 2) was developed in discussion with a Dunghutti artist and members of the project team. The team reflected on the policy, strategic and research base for the project; findings from the literature review and yarning groups; and their own experiences and knowledges working with Aboriginal communities. These reflections were sketched onto large sheets of paper, organised into categories, and mapped back to the core artwork. After four iterations of this process, the artist converted the design into the diagram for further consultation.

### 2.4. Phase 3: Scoping Literature Review

Our scoping review [33] found three central intersectional themes to embedding cultural safety in health professional child protection responses for Aboriginal children in hospital settings. These themes were: relationships; organisational processes; and culture. Safe cultural governance lay over the intersection of these three themes. The scoping review search was unable to identify any published articles that specifically addressed the intersection of Aboriginal identity, cultural safety, cultural competence and child protection in the hospital setting. Nor was there any specific translational framework available to guide practitioners to develop competence in delivering culturally safe responses in this intersecting context. These findings went on to inform the approach to qualitative data collection and the generation of original evidence.

### 2.5. Phase 4: Yarning Groups and In-Depth Interviews

A summary of the qualitative component of this project is presented below, with detailed description of the qualitative data collection, analysis and findings to be published elsewhere. A cultural strengths-based approach requires genuine and safe cultural governance processes [33,52] and respect for the oral tradition of yarning [58,59,60,61]. Respecting the cultural diversity of Aboriginal Australians meant the development and utilisation of a culturally safe yarning group protocol. This informed selection of male or female facilitators known (or not known) to the participants, the gender composition of the yarning groups, and the Aboriginality of the facilitator.

Yarns were conducted with a convenience sample of 27 Aboriginal health professionals and community members with linkages to Aboriginal nations from around Australia. The professional backgrounds of participants included child protection, nursing, medicine, allied health, executive management, health promotion, and early childhood education. The yarns explored how health professionals work together, and with families, to care for at-risk Aboriginal children. Five themes emerged from the yarning groups that aligned with evidence from the literature review and the experiences of Aboriginal peoples when accessing health services. These were genuine engagement and understanding; racism; historical bias; equity; and culture.

### 2.6. Phase 5: Clinical Utility Testing

Nine in-service sessions were delivered to present the proposed model and evaluate the clinical utility with 96 staff members across the four participating hospitals. The project team designed a paper-based survey to evaluate the model utility and appropriateness, and to identify potential barriers and enablers to model uptake from the perspective of clinicians in the field.

The survey comprised six multiple choice items for participants to rate the usefulness of the model, and the anticipated ease of implementing the principles into practice on a Likert scale from one to five. The participant’s confidence in applying the principles to practice was measured on a Likert scale from one to four. The five short answer response questions invited respondents to identify potential barriers and enablers to implementation and potential benefits to both health services and Aboriginal families.

The clinical utility survey was completed by 60 respondents: 57 clinicians who attended one of the in-service sessions and three others who were Aboriginal members of the project steering committee. The quantitative data were summarised as descriptive statistics, and qualitative findings were analysed for recurring themes and incorporation into the final model design implementation plan.

## 3. Results

The results of the five project phases of Daalbirrwirr Gamambigu and its components are described here. The model is a practical, 25-page document designed for use by frontline clinicians, their managers, and hospital executives to guide the delivery of culturally safe child protection responses for Aboriginal families in hospital settings. The visual elements of the model document design reflect the value of Aboriginal Australian artwork in promoting patient engagement in the design of healthcare settings [62], and accords with health policy for creating welcoming hospital environments [63].

The model is designed in such a way that it presents all the elements of a culturally safe patient journey. These elements are: (I) the thematic diagram of the model; (II) the use of clinical yarning as a tool for communication with families; and (III) a culturally adapted version of ISBAR for interprofessional communication. A real-world example of these elements is provided in “Latrell’s Story”, a fictional scenario to guide clinicians in the practical application of the model. Additional resources include: a cultural safety checklist for clinicians; an index of resources for Aboriginal families; and the Daalbirrwirr Gamambigu Aboriginal Consultation Diagram (Figure 1). A guide to organisational model implementation and monitoring is provided in an accompanying document.

### 3.1. Thematic Diagram and a Culturally Safe Patient Journey

**Figure 3:**
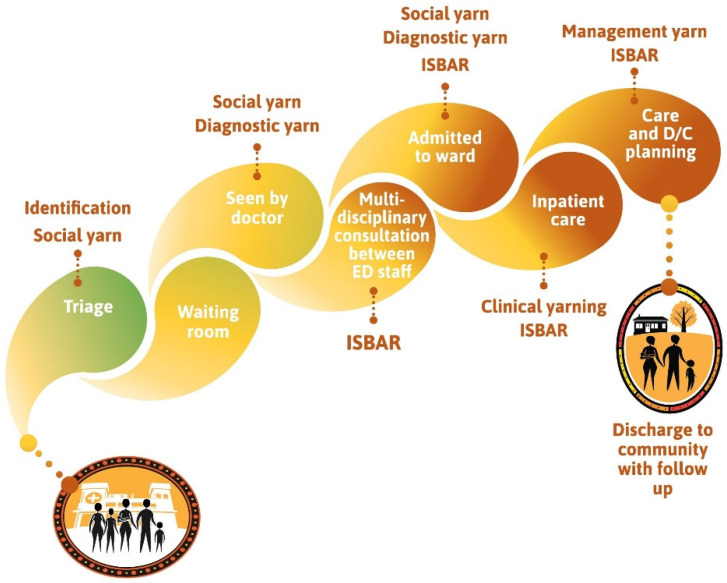
Culturally safe patient journey.

The thematic diagram (Figure 2) forms the foundation of the model, is rooted in strong Aboriginal cultures, and is one that has thorough community engagement embedded in all health service organisational processes. The child is of primary importance, and grows up supported by community, family, and Elders. When applied to clinical practice with clinical yarning and a culturally adapted ISBAR, it reflects the aim of the model for Aboriginal Australian families; which is to ensure these families experience respect, dignity and empowerment in their patient journey (Figure 3). The culturally safe patient journey illustrates the points and pathways of the hospital journey where Aboriginal families can exert influence, and where hospital staff can acknowledge their roles in listening and learning with respect, and their roles in advocating for Aboriginal families.

The Daalbirrwirr Gamambigu thematic diagram (Figure 2) shows:

- The child is at the centre of care and grows up supported by community, family, and Elders (following [2]).
- The many outer contextual factors are depicted as “yellow balls’” and are inspired by the flowers of the Australian tree *Acacia Jennerae.*
- A ‘tree of life’ inspired by the trauma-informed perspective of the collective healing tree for Stolen Generations members and their descendants [64].
- Thematic nests from yarning groups such as family, community and Elders, reflecting the strengths in the cultural roots of life.
- The critical success factors (following [45]) of cultural safety are shown as building on a foundation of community engagement, strong Aboriginal cultures and safe health services.

In phase 5 (clinical utility testing), 72% of the 57 in-service participants felt the approach would be *very* to *extremely useful* and 95% thought it could be *easily* or *moderately challenging to apply* in practice.

### 3.2. Clinical Yarning

**Figure 4:**
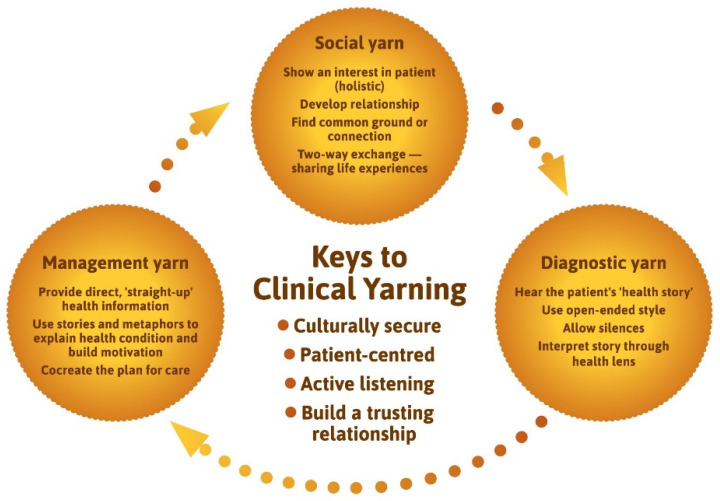
Keys to clinical yarning (adapted from Lin, Green and Bessarab (2016)).

The clinical yarning model (Figure 4) was adapted from the work of Lin, Green and Bessarab (2016) and illustrates the key elements to effective communication between health professionals and Australian Aboriginal families [65].

For the clinical yarn, a process was adopted based on extant research where clinicians, stakeholders, and patients came together to yarn through the relevance of the Lin et al. (2016) model. The ‘key elements of clinical yarning’ were slightly adapted in response to stakeholder feedback and redrawn to fit within the artistic design of the model document.

Clinical yarning guides clinicians to find common ground or connection with families through two-way exchange and the sharing of experiences in a Social Yarn. Following this, conversation transitions to a diagnostic yarn, in which open-ended questions and long silences are used to hear the patient’s health story, which is then interpreted through a biomedical (or child safety) lens. In the management yarn the clinician provides “straight-up” health information and critically cocreates a plan for care. When tested for clinical utility, almost three quarters (73%) of 57 in-service participants thought clinical yarning would be *very* or *extremely useful.*

### 3.3. Culturally Adapted ISBAR

**Figure 5:**
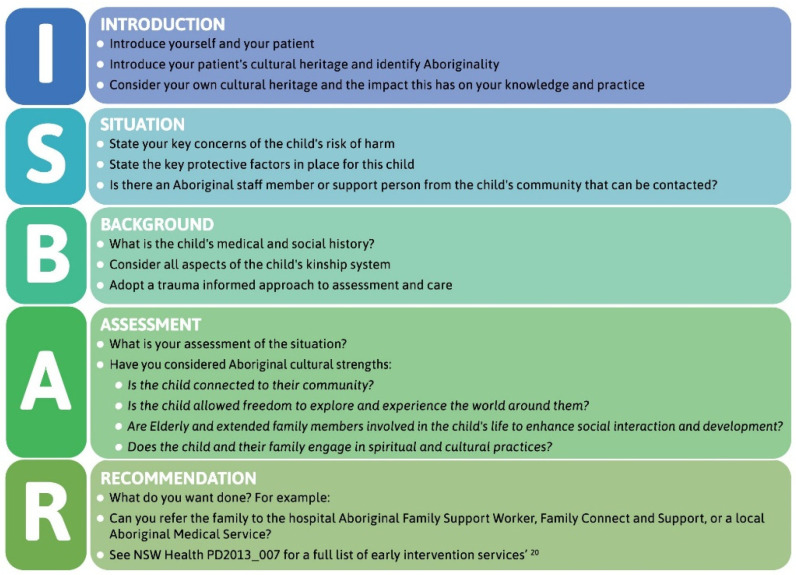
Culturally adapted ISBAR.

Study site clinicians routinely conduct clinical handover following the ISBAR mnemonic [66], where ISBAR is a tool that standardises this routine process while leaving room for situational variation. When a health professional suspects an Aboriginal child is at risk of harm, the model provides a culturally adapted version of this approach that follows the standardised process of ISBAR, while incorporating knowledge of Aboriginal strengths and family culture (Figure 5).

The culturally adapted ISBAR template is a concise, stepwise guide to presenting timely and relevant medical and cultural information in routine professional communications. In clinical utility testing, almost three quarters (71%) of 57 in-service participants thought the modified ISBAR would be *very* or *extremely useful*.

### 3.4. Cultural Safety Checklist for Clinicians

**Figure 6:**
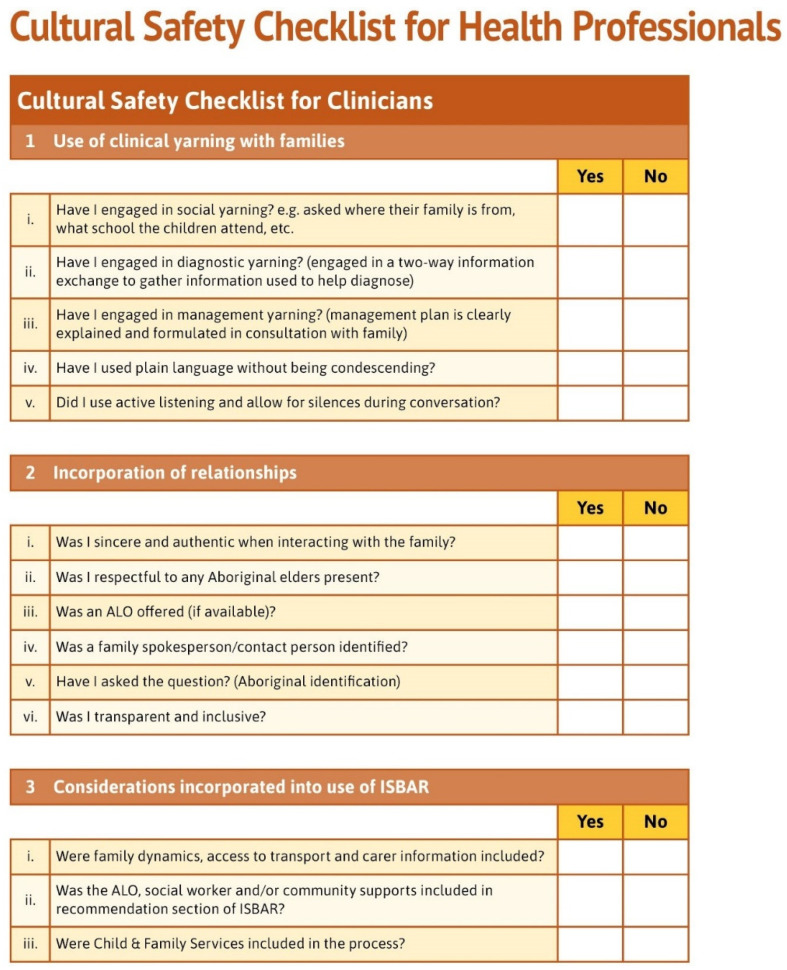
Cultural safety checklist for clinicians.

Clinical stakeholders and participants in the clinical utility testing identified the need for a self-assessment checklist to guide (but not dominate) their practice with Aboriginal families. In response, the Cultural Safety Checklist for Clinicians was developed for use as a wallet-sized prompt card for attachment to staff identification badges, as large-scale posters for display in clinical areas, and for in staff education inductions (Figure 6). The checklist prompts clinicians to reflect on use of core model elements in clinical yarning with families; the incorporation of relationships; and considerations in the use of ISBAR.

### 3.5. Clinical Utility Testing of the Model

The model was rated as very to extremely useful by 72% (*n* = 43) of respondents (Mean 3.8/5), 73% (*n* = 44) rated clinical yarning as very to extremely useful (Mean 4/5), and 72% (*n* = 43) rated the culturally adapted ISBAR as very to extremely useful (Mean 3.9/5).

Of the 100% (*n* = 60) of respondents who felt that the model could be implemented into clinical practice (Mean 3.3/4), 33% (*n* = 20) thought it could be easily implemented, 62% (*n* = 37) thought it could be implemented but would be moderately challenging, and 5% (*n* = 3) thought it could be implemented by would be very challenging. Perceived confidence levels varied with 30% (*n* = 18) of respondents very or extremely confident to apply the model, and 70% (*n* = 42) somewhat or slightly confident to apply the model.

## 4. Discussion

The aim of Daalbirrwirr Gamambigu is to guide clinical and organisational approaches to a culturally safe patient journey. It does this through a detailed framework with its core elements of clinical yarning with Aboriginal families; a culturally adapted ISBAR for interprofessional communication; a clinician checklist; and culturally designed resources. Rigorous community and clinician consultation attests to the potential of Daalbirrwirr Gamambigu to be successfully implemented and integrated into existing health and child protection services, including policy directions. Thus far, we have tested its clinical utility, and the next step is to use implementation science to evaluate effects and outcomes. To date, there has been a void in this space which has led to at-risk Aboriginal families becoming engaged with child protection services receiving inappropriate and insufficient healthcare. The model is one tool that may serve to turn around the reluctance of a non-Aboriginal health workforce to engage with cultural awareness and respect because it provides a practical guide for clinicians to follow. This may not only result in improved referral to support services but also reduce the impact of serious longer-term outcomes of referral to child protection services for, in particular, neglect [67].

### 4.1. The Daalbirrwirr Gamambigu (Safe Children) Model of Care

Cultural safety is relevant at every ‘level’ of care [27,33,34,45], and the model thematically interconnects organisational types and levels of cultural safety (Figure 2). The culturally adapted tools prioritise honesty and authenticity in health professional practice and contribute to dismantling institutional racism with safe organisational processes. This aligns with the concept of cultural support [68], a key concept in child protection policy.

### 4.2. Appropriate Governance and Cultural Engagement

Phases 1 and 2 of the project involved the establishment of cultural governance, project oversight, cultural engagement processes, and mapping. Positive cultural factors are present in each level of intervention (child and/or family) and the service system itself [27], and is acknowledged in related models, for example the Koorliny Mort and Wadja New models of care [69,70]. Respecting the diversity of the more than 500 Aboriginal nations in Australia is key to safe cultural governance and implementation [71]. While the model demonstrated strong cultural acceptance among Aboriginal stakeholders in the study sites and participating organisations, consideration of cultural diversity will need to be continued in implementation through strengthening and extending these relationships.

The five project phases and quality appraisal (Appendix A) [72] demonstrate the degree of cultural rigor [73] in the model development. In the absence of detailed explorations of cultural rigor in Australian research ethics, our approach aligns with international ethics processes [74] so that Aboriginal Australians were involved in the design, governance, management, implementation and analysis of the research. This culturally respectful research process, an enabling policy context, and enthusiastic clinician engagement in the topic of cultural safety, meant that the project process and relational methodology was decidedly non-linear. This is a reflection of the importance of building relationships as the foundation for practice [35], and locates the model as a point of reference for intersectoral approaches to care. The model bridges the gaps between child maltreatment policy, research and practice [75], and serves as an aide to overcoming the fragmentation in service provision to Aboriginal families [36].

### 4.3. A Culturally Safe Patient Journey

Health professionals who want to ‘step-up’ [76], and build a critical consciousness [77] beyond that of cultural awareness training [78], can draw on the model to restructure their practice to embed Aboriginal worldviews [79]. The graphic of the culturally safe patient journey (Figure 3) is an original concept by an Aboriginal project officer, and culturally connects the visual, cognitive, social and pedagogic systems [80]. In that journey, Aboriginal yarning enables honest, respectful and clear communication with families, which is key to the provision of safe and effective clinical care [33]. Implementation of the model throughout the patient journey puts the importance of taking time [34,35,81] to build trust [59,82] in forming meaningful therapeutic relationships [83,84] with Aboriginal Australians into a practical format.

### 4.4. Clinical Yarning

A key clinical resource in the model was the adaptation of clinical yarning as a method for building relationships of trust [65]. The Daalbirrwirr Gamambigu thematic diagram (Figure 2) and the yarning group data analysis acknowledge the importance of power (a key dimension of cultural safety) in spaces where healthcare and child protection services intersect. Language builds meaning [85], and communication between clinicians and Aboriginal children and families defines the way we think about the child and the family.

A key policy lever challenging institutional power is the embedding of cultural safety in Australian healthcare standards [86], which may influence a shift within the dominant discourse in health and child protection services from that of the clinician to that of the Aboriginal child, family and community [8]. The project was also supported by cultural plans local to the study sites [41,87], locating the model as a ground level clinical strategy to remove the culturally blind [88] filter that is placed over Aboriginal families’ culture and experiences. As we have argued elsewhere [33], embedding cultural safety means that the patient has the power to determine what is a culturally safe practice; professionals reflect on their personal and professional power in care; and services respond to cultural differences.

The language used by clinicians in hospital settings shapes the interactions they have with Aboriginal families [89]. That is, institutional discourse occurs in an organisational setting where the clinician is the expert driving the discourse. Communication is a key factor in creating safe environments for Aboriginal patients [90]. Tanner argued that “communication difficulties exacerbate knowledge and power differentials for many families” [32] and Jennings emphasised that talk played a pivotal role in “mediating the power differentials between health professionals and Indigenous clients” [89]. Communication between clinicians and non-Aboriginal and Aboriginal people defines the way clinicians think about the child and family. We aim to remove the filter placed over culture and experiences, and to shift the dominant discourse in health and child protection services from that of the clinician to that of the Aboriginal child, family and community.

### 4.5. Culturally Adapted ISBAR

The model aligns with policy that calls for culturally safe care to be delivered in regard to cultural identity [91,92] and in response to peoples’ cultural uniqueness [93,94,95]. The culturally adapted ISBAR tool [66] prompts communication of the cultural diversity and strengths of Australian Aboriginal families and culture (Figure 4). The mediation of power differentials—evident in clinical yarning—is also central to the content of the culturally adapted ISBAR. This approach advances culturally responsive communication research [90] by demonstrating how communication between two worlds can be practically integrated in interprofessional communication through an enabling workplace [96]. Such processes are critical to address the widespread inadequacies in communications between health organisations and with Aboriginal families and the subsequent effects on engagement, service coordination, and continuity of care [97]. In going beyond the communication skills developed in standard cultural awareness training [98,99], health and child protection professionals may utilise these techniques as common points of reference for quality care.

### 4.6. Daalbirrwirr Gamambigu at the Intersection of Public Health and Child Protection

In the child protection sector, numerous reports, policies and strategies call for reforms. For example, the national level Fourth Action Plan of the National Framework for Protecting Australia’s Children [100] has a priority to improve outcomes for Aboriginal children and improving the application of the Aboriginal and Torres Strait Islander Child Placement Principle [101]. The Family Matters Report 2020 calls for a dedicated national strategy to implement national standards of practice for child protection [39] and the Australian Government’s ‘National Agreement on Closing the Gap’ includes an outcome to reduce over-representation in the child protection system [102]. The potential significance of the model lies at the intersection of healthcare and child protection systems because both systems have enabling policy environments that emphasise cultural safety where the cultures of Aboriginal peoples:

- matter in health and wellbeing policy [102,103,104];
- can be embedded in health professional accreditation [105];
- are a priority in child safe organisations [106,107];
- can enhance culturally respectful and appropriate emergency department team skills [108];
- influence emergency department care [109];
- inform interdisciplinary approaches to child care [110];
- are a foundation for cultural training for health professionals [111];
- lead to the provision of care that is judged to be culturally safe [112]; and
- can help improve the effectiveness of communication with Australian Aboriginal families [113].

### 4.7. Strengths and Limitations

The Daalbirrwirr Gamambigu Model is the first empirical resource to support clinicians and organisations to engage with at-risk Aboriginal families in Australian hospitals and improve outcomes through referral to appropriately targeted early intervention services. It is the first Australian translational action research project to clearly articulate how to create cultural safety in research and in practice since the definition was noted as an emerging policy concept in hospital care in early 2000 [37].

There is a major gap in our knowledge of how Aboriginal families use hospital and other mainstream health services, and how this utilisation and engagement is affected by better health professional communication and innovative models of care. For example, some Aboriginal families do not identify as such on presentation to hospital for personal or historical experiences of racism and trauma from government institutions. The extrapolation of findings from the qualitative study was limited to some extent by recruiting a convenience sample to the yarning groups. A broad and more diverse representation from Aboriginal nations throughout New South Wales is recommended for future research.

The project gave rigorous attention to cultural forms of engagement and communication by providing a practical guide for interprofessional collaboration in hospitals. The project also contributed to reframing the nature of interaction that is currently determined by western institutional discourse.

Known barriers to translation of research findings into practice were addressed through early and ongoing engagement with community stakeholders and ministerial policy makers. Study limitations and potential barriers to implementation identified in clinical utility testing will be addressed in the feasibility stage of translation. This approach will support clinicians and organisations with required learnings and processes, engaging with a broader range of Aboriginal stakeholders and the use of patient experience measures as a key domain of evaluation.

## 5. Conclusions

The Daalbirrwirr Gamambigu Model of Care is a practical link between policy and daily practice that shows how staff systems in mainstream organisations can be disrupted to decolonise professional power and institutional racism. In doing so, the model can be used as a tool to support and advocate for culturally safe health services for Aboriginal Australian families. If cultural safety can be successfully embedded using our model within these systems and integrated into routine healthcare practice, significant improvements in Aboriginal child and family outcomes, including child protection outcomes, are possible.

## Data Availability

Not applicable.

## Appendix A

Aboriginal and Torres Strait Islander Quality Appraisal Tool [72]

1. Did the research respond to a need or priority determined by the community?
  - **Unmet need identified by research project steering committee**. The Daalbirrwirr Gamambigu Project developed from the SAFE-ED project where the steering committee members recognised the unmet need of responding to Aboriginal children at risk of harm who presented to emergency departments.
  - **Testing relevance of idea with staff**. JF and TF tested the relevance of this idea in discussions with Aboriginal and non-Aboriginal colleagues in the Mid North Coast Local Health District and the Sydney Children’s Hospital Network. JF and TF received affirmation that there was an unmet need in emergency department responses to Aboriginal children at risk of harm.
  - **Finding research funding**. JF and TF investigated opportunities for a Medical Research Future Fund (MRFF) grant entitled “Embedding cultural safety in child protection policies for Aboriginal families in NSW paediatric care settings” with the formal support of the Director of Aboriginal Health (Mid North Coast Local Health District) and Executive Aboriginal Health Manager (Sydney Children’s Hospitals Network).
  - **Engaging with Aboriginal community in local areas**. TF, a long-time community member in the local area, discussed the grant idea further with Aboriginal staff members, who provided TF with advice to contact Aboriginal community (not health staff), such as the Coffs Harbour Aboriginal Community Care Centre Inc. (known as Abcare) and the NSW Child, Family and Community Peak Aboriginal Corporation (AbSec). This engagement involved genuine contact between TF/CG and AbSec staff at many stages of the development of the Daalbirrwirr Gamambigu project. The project team are now finalising endorsement of the Framework and MoC with the AbSec CEO.
  - **Engaging with Aboriginal Researchers**. TF and JF had established the project steering committee which included a number of Aboriginal advisors at the clinical, managerial and executive level from two Local Health Districts. Furthermore, the project team included three Aboriginal project officers at various stages of the project and Aboriginal members of the research team Associate Professor Donna Hartz, Dr Mark J Lock, and Dr Liesa Clague.
  - **Engaging with Aboriginal staff**. The Aboriginal staff involved in the project are listed in an Appendix of the Framework and Model of Care documents are Clinton Gibbs, Joanne Shipp, Joseph Bryant, Robyn Martin, Amanda Ryan, Mick Scarcella, Jessica Morris, and Brenna Bernardino.
  - **Testing the idea with reference to policy, strategy, and research**. There were numerous formal published sources of information where the needs of Aboriginal families and children were noted in regards to hospital care and child protection systems. The scoping literature review demonstrates the depth of investigation that the team went to so that the project aligned with the needs of Aboriginal families as described in the formal literature.
  - **Occurs within a broader policy context**. The idea for the project occurred within an enabling policy context where the Mid North Coast Local Health District and the Sydney Children’s Hospital Network had strategies to Close the Gaps in Aboriginal health outcomes. These organisations operated in accord with NSW Government policy to reduce disadvantages experienced by Aboriginal people. In practice, an enabling organisational environment and policy context allows engagement and consultation activities to occur with Aboriginal people.
2. Was community consultation and engagement appropriately inclusive?
  - The **consultation diagram** shows the key points of engagement with stakeholders appropriate to researching the intersection between healthcare and child protection systems.
  - **Project Staff** are listed in the Framework and Model. There were two staff from the University of Sydney (lead academic organisation, one non-Aboriginal person and one Aboriginal person); five staff from the Mid North Coast Local Health District (sponsoring organisation, government health agency, four Aboriginal people and one non-Aboriginal person); three staff from the Sydney Children’s Hospitals Network (participating organisation, government health agency, one Aboriginal person and two non-Aboriginal people) and three independent staff, two Aboriginal and one non-Aboriginal. Of the ten project staff, seven were Aboriginal and three were non-Aboriginal peoples. Career spans show that six Aboriginal staff (CG, ML, LC, JS, JB, JM) combined had careers in Aboriginal affairs. Career spans for the two non-Aboriginal staff (JF and TF) were developed in paediatric/midwifery nursing practice, child protection and research, with a high awareness of Aboriginal peoples’ needs in healthcare.
  - **AbSec Conference Workshop Participants**: There were over 50 participants in the workshop that took place in Coffs Harbour on Gumbaynggirr Country in November 2019. This was a culturally appropriate event because of the conference theme (Strong Communities Strong Kids), it was hosted by the AbSec (the NSW Child and Family Peak Aboriginal Corporation), and the conference provided participants with an opportunity to strengthen their skills and knowledge in supporting Aboriginal children, young people and families. The location (Coffs Harbour) was also the site of a sponsoring organisation (the Mid North Coast Local Health District); and the participants were Aboriginal stakeholders with professional and personal interests in the Daalbirrwirr Gamambigu project. The structure of the workshop was designed around circles of yarning, with three roundtable topics (clinical yarning, model of care, and ISBAR). The workshop comments and suggestions informed the implementation of the project.
  - **Sydney Children’s Hospital Network Staff (SCHN)**: There were nine staff from the SCHN, including the Chief Investigator (JF, non-Aboriginal), child protection unit director (SM, non-Aboriginal), executive medical director (MM, non-Aboriginal), staff specialist (DT, non-Aboriginal), diversity health coordinator (JC, non-Aboriginal), senior nursing research fellow (SSL, non-Aboriginal), director of nursing and midwifery education (SW, non-Aboriginal), director of clinical integration (MD, non-Aboriginal), and the Aboriginal health management advisor (MS, Aboriginal). Of the nine SCHN members, one is Aboriginal and ten are non-Aboriginal. Their roles and careers are in the fields of nursing, medicine, clinical, information technology, paediatrics, specialists, research, and management. The SCHN members were active in health and child protection areas.
  - **Aboriginal organisations**. Five Aboriginal organisations participated in this project. The Aboriginal Health and Medical Research Council (AH and MRC, the peak advocacy body and Human Research Ethics Committee for Aboriginal community controlled health organisations in NSW); AbSec, the NSW Child and Family Peak Aboriginal Corporation (lead advocacy organisation for Aboriginal child protection in NSW); AbCare (Aboriginal Children/Young People in Out-of-Home-Care, Coffs Harbour, NSW) is the lead organisation in the Mid North Coast Local Health District that provides services for Aboriginal people in out-of-home-care to Aboriginal communities in Coffs Harbour, Bellingen and Clarence Valley areas, the Secretariat National Aboriginal; and Torres Strait Islander Child Care (SNAICC is the national peak body for Aboriginal child protection, of which AbSec is a member); the Congress of Aboriginal and Torres Strait Islander Nurses and Midwives (CATSINaM) is the national peak professional association for Aboriginal and Torres Strait Islander Nurses. These organisations represent Aboriginal communities and professionals in child protection and health care, from the local level (AbCare), to state level (AbSec, AH&MRC), to the national level (SNAICC, CATSINaM).
3. Did the research have Aboriginal and Torres Strait Islander research leadership?
  - The research was not led by Aboriginal researchers. However, Aboriginal researchers were integral to its evolution and development through the governance committee, research assistance, research advice, and in technical aspects of the project such as ethics applications, yarning groups, interviews, conference presentations, data analysis, and writing (ML, LC, CG, JS, JB, MS).
4. Did the research have Aboriginal and Torres Strait Islander governance?
  - The Daalbirrwirr Gamambigu project had a Steering Committee of 21 members representing the three stakeholder organisations: the University of Sydney, the Mid North Coast Local Health District, and the Sydney Children’s Hospital Network. The steering committee had Aboriginal (*n* = 6) and non-Aboriginal (*n* = 15) members and was chaired by a non-Aboriginal Executive chairperson.
5. Were local community protocols respected and followed?
  - The protocols for local community engagement were learned through oral communication with Aboriginal people, as cultural authority is learned and understood through the developing of trusting relationships. In this project, the local communities were not only Australian Aboriginal communities but also the communities of practice established around child protection in healthcare settings. Some examples will explain. The Aboriginal staff of the MNCLHD (e.g., RM, AR, CG, JS, and JB) have 75 years of living and working with Aboriginal people throughout the Mid North Coast Local Health District. Key stakeholders in each of the Aboriginal organisations (SNAICC, AbSec, and AbCare) provided direction on community engagement within their respective networks to TF. The degree and extent of engagement, as aligned with local community protocols, was not systematically documented with respect to cultural protocols or yarning and oral knowledge transfer.
6. Did the researchers negotiate agreements in regards to rights of access to Aboriginal and Torres Strait Islander peoples’ existing intellectual and cultural property?
  - This was not explicitly negotiated but will be considered in the evaluation phase.
7. Did the researchers negotiate agreements to protect Aboriginal and Torres Strait Islander peoples’ ownership of intellectual and cultural property created through the research?
  - This was not explicitly negotiated.
8. Did Aboriginal and Torres Strait Islander peoples and communities have control over the collection and management of research materials?
  - The data collection and materials are owned and managed by the University of Sydney, and stored in a secure digitally encrypted location. The control and management of these materials was overseen by the steering committee and administered by the research team. Data collection, analysis and publication was undertaken by Aboriginal and non-Aboriginal staff.
9. Was the research guided by an Australian Aboriginal research paradigm?
  - The methodology was founded on the combination of the Wiradjuri developed Ngaa-bi-nya program evaluation framework and the Western i-PARIHS framework in keeping with the Australian ethic of cultural safety being a shared learning experience.
  - The methodology was intersectional in acknowledgement of the cultural interface (following Nakata) between Aboriginal and Western world views as this is reflected in the empirical methodology of data collection such as the scoping literature review and yarning groups.
  - The ethic of the project was founded on enabling cultural safety in every point and pathway of healthcare governance which is stated in the Mid North Coast Aboriginal Health Authority endorsed Aboriginal Cultural Safety and Security Framework.
10. Does the research take a strengths-based approach, acknowledging and moving beyond practices that have harmed Aboriginal and Torres Strait peoples in the past?
  - The Daalbirrwirr Gamambigu project privileged the cultural strengths and cultural voices of Aboriginal peoples. This is evidenced in the philosophy informing the project (cultural safety), the methodology (Ngaa-bi-nya, i-PARIHS, Cultural Respect Framework for Aboriginal and Torres Strait Islander Health, and the Family Matters Roadmap), the consultation process which involved many Aboriginal people and Aboriginal organisations (who then formally endorsed the Daalbirrwirr Gamambigu materials), the governance committee, feedback to stakeholders and broader audiences, all aspects of the data analysis and writing, commissioning of the artwork from a local Aboriginal artist, and the use of an Aboriginal language through engagement with an Muurrbay Aboriginal Language and Culture Co-operative.
11. Did the researchers plan to and translate the findings into sustainable changes in policy and/or practice?
  - The methodology, informed by both Ngaa-bi-nya and i-PARIHS, was directed towards translational research that benefits Aboriginal families and the professionals who work with them. The Daalbirrwirr Gamambigu Framework and Model will be cited in policy documents (e.g., NSW Aboriginal Health Plan) as direct evidence of cultural safety research. It will be referenced in peer reviewed journal articles in line with the need to produce a high quality evidence for policy and practice. It was developed into the training resources (the Framework and Model) after extensive stakeholder consultation and practitioner workshops. In 2022, the Daalbirrwirr Gamambigu Model will be trialled and evaluated in both the Sydney Children’s Hospital Network (metropolitan) and the Mid North Coast Local Health District (regional).
12. Did the research benefit the participants and Aboriginal and Torres Strait Islander communities?
  - The benefit is formally demonstrated in the Aboriginal organisational endorsement of the Daalbirrwirr Gamambigu project. This endorsement underscores the determination of the research team to ensure meaningful consultation with Aboriginal stakeholders as a key way to determine if the benefit is seen by Aboriginal people.
13. Did the research demonstrate capacity strengthening for Aboriginal and Torres Strait Islander individuals?
  - There were many Aboriginal people involved in this project from clinical experts, executive officers, project workers, researchers, staff members, and from Aboriginal community organisations. The strengthening is about participation in a formal research translation activity, paid employment as project officers, paid employment as researchers, inclusion in data analysis, inclusion in publications, and inclusion in conference and workshop activities. That is capacity building was evident in employment, research participation, formal publication, and communication and engagement activities. Three Aboriginal project officers employed across the project duration have since gone on to permanent roles in positions of seniority in government health organisations.
14. Did everyone involved in the research have opportunities to learn from each other?
  - We learned from each other as members of the governance committee (how to translate research into practice and Aboriginal community benefit); in communication and engagement activities (workshop design and conduct); in data analysis and writing (collecting data, interpreting data, and writing in formal reports and journal articles); and in routine meetings between non-Aboriginal and Aboriginal researchers.
